# Vaginal versus Obstetric Infection *Escherichia coli* Isolates among Pregnant Women: Antimicrobial Resistance and Genetic Virulence Profile

**DOI:** 10.1371/journal.pone.0146531

**Published:** 2016-01-19

**Authors:** Emma Sáez-López, Elisabet Guiral, Dietmar Fernández-Orth, Sonia Villanueva, Anna Goncé, Marta López, Irene Teixidó, Anna Pericot, Francesc Figueras, Montse Palacio, Teresa Cobo, Jordi Bosch, Sara M. Soto

**Affiliations:** 1 Department of Microbiology, Hospital Clínic - Universitat de Barcelona, Barcelona, Spain; 2 ISGlobal, Barcelona Ctr. Int. Health Res. (CRESIB), Hospital Clínic - Universitat de Barcelona, Barcelona, Spain; 3 BCNatal-Barcelona Center of Maternal-Fetal and Neonatal Medicine (Hospital Clínic and Hospital Sant Joan de Déu), Barcelona, Spain; 4 Institut D´Investigacions Biomèdiques August Pi I Sunyer (DIBAPS), Barcelona, Spain; 5 University of Barcelona, Barcelona, Spain; 6 Center for Biomedical Research on Rare Diseases (CIBER-ER), Barcelona, Spain; State University of Maringá/Universidade Estadual de Maringá, BRAZIL

## Abstract

Vaginal *Escherichia coli* colonization is related to obstetric infections and the consequent development of infections in newborns. Ampicillin resistance among *E*. *coli* strains is increasing, which is the main choice for treating empirically many obstetric and neonatal infections. Vaginal *E*. *coli* strains are very similar to extraintestinal pathogenic *E*. *coli* with regards to the virulence factors and the belonging to phylogroup B2. We studied the antimicrobial resistance and the genetic virulence profile of 82 *E*. *coli* isolates from 638 vaginal samples and 63 isolated from endometrial aspirate, placental and amniotic fluid samples from pregnant women with obstetric infections. The prevalence of *E*. *coli* in the vaginal samples was 13%, which was significant among women with associated risk factors during pregnancy, especially premature preterm rupture of membranes (p<0.0001). Sixty-five percent of the strains were ampicillin-resistant. The *E*. *coli* isolates causing obstetric infections showed higher resistance levels than vaginal isolates, particularly for gentamicin (p = 0.001). The most prevalent virulence factor genes were those related to the iron uptake systems revealing clear targets for interventions. More than 50% of the isolates belonged to the virulent B2 group possessing the highest number of virulence factor genes. The ampicillin-resistant isolates had high number of virulence factors primarily related to pathogenicity islands, and the remarkable gentamicin resistance in *E*. *coli* isolates from women presenting obstetric infections, the choice of the most appropriate empiric treatment and clinical management of pregnant women and neonates should be carefully made. Taking into account host-susceptibility, the heterogeneity of *E*. *coli* due to evolution over time and the geographical area, characterization of *E*. *coli* isolates colonizing the vagina and causing obstetric infections in different regions may help to develop interventions and avoid the aetiological link between maternal carriage and obstetric and subsequent puerperal infections.

## Introduction

*Escherichia coli* are reported as one of the most common organisms found in the genital tract of non-pregnant (9–28%) and pregnant women (24–31%). Vaginal *E*. *coli* (VEC) strains are considered to be a reservoir for vaginal and/or endocervical colonization in pregnant women, and an important step in the development of urinary tract, intra-amniotic and puerperal infections through ‘fecal-vaginal-urinary/neonatal’ transmission [1,2]. Histologically, subclinically or clinically diagnosed chorioamnionitis is an obstetric infection caused principally by ascending microorganisms from the vagina which may lead to maternal or foetal complications, including postpartum endometritis, bacteraemia or sepsis. *E*. *coli* is considered to be normally involved in these infections despite their polymicrobial susceptibility [3].

The most frequent antibiotic treatment for preterm rupture of membranes, suspected chorioamnionitis or neonatal sepsis and bacterial meningitis is ampicillin plus an aminoglycoside, normally gentamicin, whereas for postpartum endometritis, clindamycin or ampicillin plus gentamicin are prescribed. Nevertheless, an increase of treatment failure has been observed due to the high percentage of ampicillin resistance found among *E*. *coli* strains [4].

The status of the immune system and host susceptibility play an important role in the outcome of an infection and disease progression [5]. However, extraintestinal infection is associated with a broad range of virulence factors (VFs) including adhesins, toxins, siderophores and invasins, among others [6–8]. VEC strains share a virulence factor profile with extraintestinal pathogenic isolates of *E*. *coli* (ExPEC), which are different from commensal flora and allow them to colonize, avoid defense mechanisms and cause extra-intestinal infections [1], [5,6], [9].

Most ExPEC are assigned to the virulent phylogenetic group B2, followed by group D, whilst groups A and B1 are frequently associated with commensal strains based on the old classification created by Clermont et al. [10]. Similar results have been observed for *E*. *coli* isolates causing bacteraemia among pregnant women and urinary tract infections (UTIs) with the revised method [11–13]

Due to the selective pressure in the habitats, commensal *E*. *coli* can turn into virulent and resistant strains [14]. VEC isolates from pregnant women may be related to obstetric infections and the consequent development of infections in newborns. In addition, these strains may be resistant to empiric treatment resulting in therapeutic failure. Therefore, the aim of this study was to characterize and compare 145 *E*. *coli* isolates from vaginal samples (colonizing) and samples of women with obstetric infections in terms of antimicrobial resistance, phylotype and the virulence profile in order to determine their virulence potential.

## Materials and Methods

### Clinical Sample Collection and Isolation of *E*. *coli*

**Fig 1:**
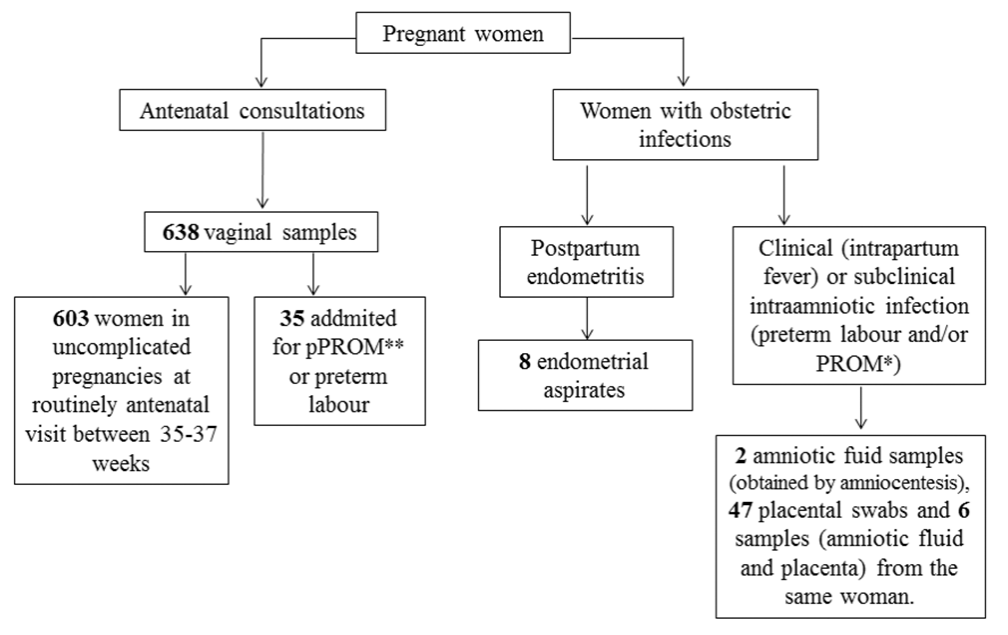
Diagram flow of the clinical sample collection. *PROM: Premature Rupture of Membranes; **pPROM: preterm PROM.

Two types of isolates were collected from pregnant women at the Maternity Fetal Medicine Department of the Hospital Clinic of Barcelona (Fig 1). On one hand, a total of 638 vaginal samples were collected on one of the antenatal visits from February 2011 to June 2013. These vaginal samples included (A) 603 from pregnant women with normal gestation and (B) 35 from pregnant women with associated risk at delivery (premature preterm rupture of membranes or preterm labour). On the other hand, 63 *E*. *coli* isolates were collected from endometrial aspirate, amniotic fluid or placenta of pregnant women with obstetric infections, including postpartum endometritis and clinical or subclinical intraamniotic infection from February 2011 to December 2014. The “Comité Ético de Investigación Clínica (CEIC)” of the Hospital Clinic of Barcelona, formed by Dr. Begoña López Pérez and Dr. Joan Albert Barberá Mir, approved the project (Ref. 2010/5720) and all women provided informed signed consent. All the samples were spread onto MacConkey Agar and incubated at 37°C overnight. Suspected colonies were confirmed by MALDI-TOF.

### Antimicrobial Resistance

Resistance profiles were determined using the standard Kirby-Bauer disk-diffusion method. The antimicrobial agents tested were ampicillin (AMP), amoxicillin/clavulanic acid (AMC), cefuroxime (CXM), cefotaxime (CTX), ceftazidime (CAZ), imipenem (IPM), tetracycline (TET), trimethoprim/ sulfamethoxazole (SXT), amikacin (AMK), gentamicin (GEN), ciprofloxacin (CIP), chloramphenicol (CHL), and fosfomycin (FOF). In addition, an Extended-Spectrum Beta-Lactamase (ESBL) confirmatory test using CTX, AMC and CAZ was carried out [15]. The results were interpreted following Clinical and Laboratory Standards Institute guidelines [16] and the *E*. *coli* ATCC25922 strain was used as the control.

### Prevalence of virulence factors genes

The virulence profile was analysed by PCR using gene-specific primers for 13 genes encoding virulence determinants usually associated with extraintestinal *E*. *coli* strains. The genes studied included: hemolysin (*hly*A), cytotoxic necrotizing factor (*cnf*1), autotrasporter toxin (*sat*1), P- fimbriae (*pap*A, *-*EF, *-*C), type 1-C fimbriae (*foc*G), heat-resistant hemagglutinin (*hra*), yersiniabactin (*fyu*A), siderophores (*iut*A and *iro*N), aerobactin (*iuc*C) and invasion of brain endothelium factor (*ibe*A) [17]. PCR was performed under the following conditions: initial denaturation at 94°C for 3 min, followed by 25 cycles of denaturation at 94°C for 30 s, the corresponding annealing temperature (55–63°) for 30 s, an extension at 72°C for 1 min and 30 s, and a final elongation at 72°C for 7 min. The amplification products were separated in 1.5% agarose gels and stained with Syber Safe. A 100-bp DNA ladder was used in each gel as a molecular size marker. Several strains previously characterized in terms of virulence in our laboratory were used as positive controls. The results were considered to be positive if the amplification product was similar to the expected molecular size.

### Phylogenetic analysis

The new and improved *E*. *coli* phylo-typing method with several modifications described by Clermont at al. [13] was used to identify *E*. *coli* isolates belonging to the eight phylogroups (B2, D, B1, A, E, Non-typeable, F, C, and E clade 1). A simplex PCR method was utilized to determine the *arp*A, *chu*A, *yja*A and TspE4.C2 genes and the concentration of the primers was 5μM instead of a quadruplex-PCR with a concentration of 20 μM.

### Statistical analysis

Statistical analyses were performed using IBM SPSS Statistics software for Windows, version 20.0. Comparisons of associations between antibiotic susceptibility, the phylogenetic group and the presence of virulence factor genes (VFGs) were made using the chi-square test when all the expected cell frequencies were ≥ 5, the chi-square with Yates’ correction when the number of expected cases was between 3 (inclusive) and 5 and the Fisher’s exact test when the number of expected cases was below 3 in at least one group. A p-value below 0.05 was considered statistically significant and p-values below 0.01 were highly significant.

## Results

### Prevalence of *E*.*coli* among the vaginal samples

Among the 638 vaginal samples collected, 82 isolates (13%) were positive for *E*. *coli*, and this was statistically or highly significant among women belonging to group B compared to group A (n = 13, 37% *versus* n = 69, 11% respectively; p = 0.0001) (Table 1). In addition, premature preterm rupture of membranes (pPROM) was the most prevalent risk factor (n = 12, 92%) among pregnant women with infection by *E*. *coli*.

**Table 1:** Prevalence of *Escherichia coli* among vaginal samples from pregnant women with normal gestation (A) and some risks at birth (B).

Pregnant women	Presence of *E*. *coli*	Absence of *E*. *coli*	Total
(A) with normal gestation	69 (11)	534 (89)	603
(B) with some risks at birth	**13 (37)** **	22 (63)	35
Total	**82 (13)**	556 (87)	638

Data are shown as no. (%).

**p-value: p = 0.000015

### Antimicrobial susceptibility

**Fig 2:**
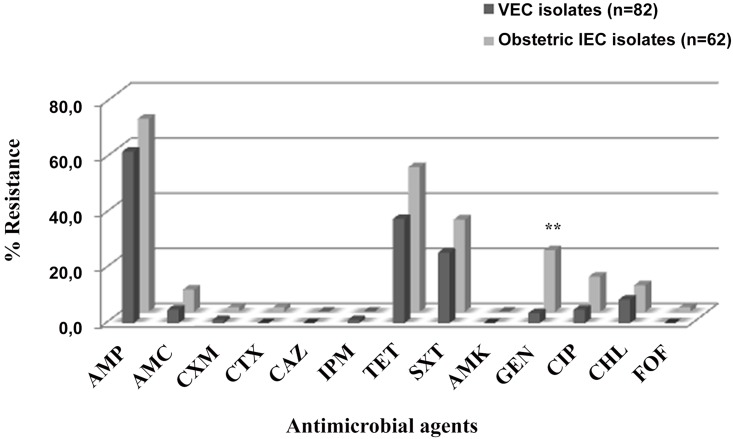
Distribution of antimicrobial resistance among VEC and obstetric IEC isolates. VEC isolates: vaginal *Escherichia coli* isolates; Obstetric IEC isolates: obstetric infection *Escherichia coli* isolates. AMP, ampicillin; AMC, amoxicillin/clavulanic acid; CXM, cefuroxime, CTX, cefotaxime; CAZ, ceftazidime; IPM, imipenem; TET, tetracycline, SXT, trimethoprim/ sulfamethoxazole; AMK, amikacin; GEN, gentamicin; CHL, chloramphenicol; CIP, ciprofloxacin and FOF, fosfomycin. (**p-value = 0.001).

Sixteen obstetric infection *E*. *coli* (obstetric IEC) (25%) and 29 VEC isolates (5%) were susceptible to all the antimicrobial agents tested. Ninety-five (65.5%) of all the isolates were resistant to ampicillin. Obstetric IEC isolates showed higher percentages of resistance to all the antibiotics than VEC isolates except for imipenem (only one vaginal resistant isolate), which was highly significant for gentamicin (22.2% *versus* 3.7%, respectively; p = 0.001) (Fig 2). The presence of ESBLs was only found in one isolate from a pregnant woman with puerperal endometritis harbouring the CTX-M-15 enzyme.

### Prevalence of virulence factor genes

Among all the isolates, the most prevalent VFGs were those related to the iron uptake systems: *fyu*A (79.3%), *iuc*C (55.9%) and *iut*A (53.8%) (Table 2). No significant differences were found between obstetric IEC and VEC isolates apart from invasin *ibe*A which was more frequently found among VEC isolates (n = 13, 15.9% *versus* n = 2, 3.2%, p = 0.014). The VFG less frequently found in all the isolates was *foc*G (VEC: 11%, obstetric IEC: 6%).

**Table 2:** Prevalence of virulence factor genes (VFGs) according to the type of isolate.

	Type of isolate		
VFGs^a^	VEC^b^ (n = 82)	Obstetric IEC^c^ (n = 63)	Total (n = 145)
*hly*A	19 (23)	12 (19)	31 (21)
*cnf*1	18 (22)	16 (25)	34 (23)
*sat*1	29 (35)	27 (43)	56 (39)
*pap*A	23 (28)	28 (44)	51 (35)
*pap*EF	21 (26)	17 (27)	38 (26)
*pap*C	20 (24)	24 (38)	44 (30)
*foc*G	9 (11)	4 (6)	13 (9)
*hra*	29 (35)	22 (35)	51 (35)
*fyu*A	66 (80)	48 (76)	114 (79)
*iut*A	37 (45)	40 (63.5)	77 (53)
*iro*N	29 (35)	23 (37)	52 (36)
*iuc*C	41 (50)	39 (62)	80 (55)
*ibe*A	13 (16)*	**2 (3)**	15 (10)

^a^VFGs: Virulence Factor Genes

^b^VEC: Vaginal *Escherichia coli*

^c^Obstetric IEC: Obstetric Infection *Escherichia coli*

*p-value = 0.014

### Phylogenetic group distribution

**Fig 3:**
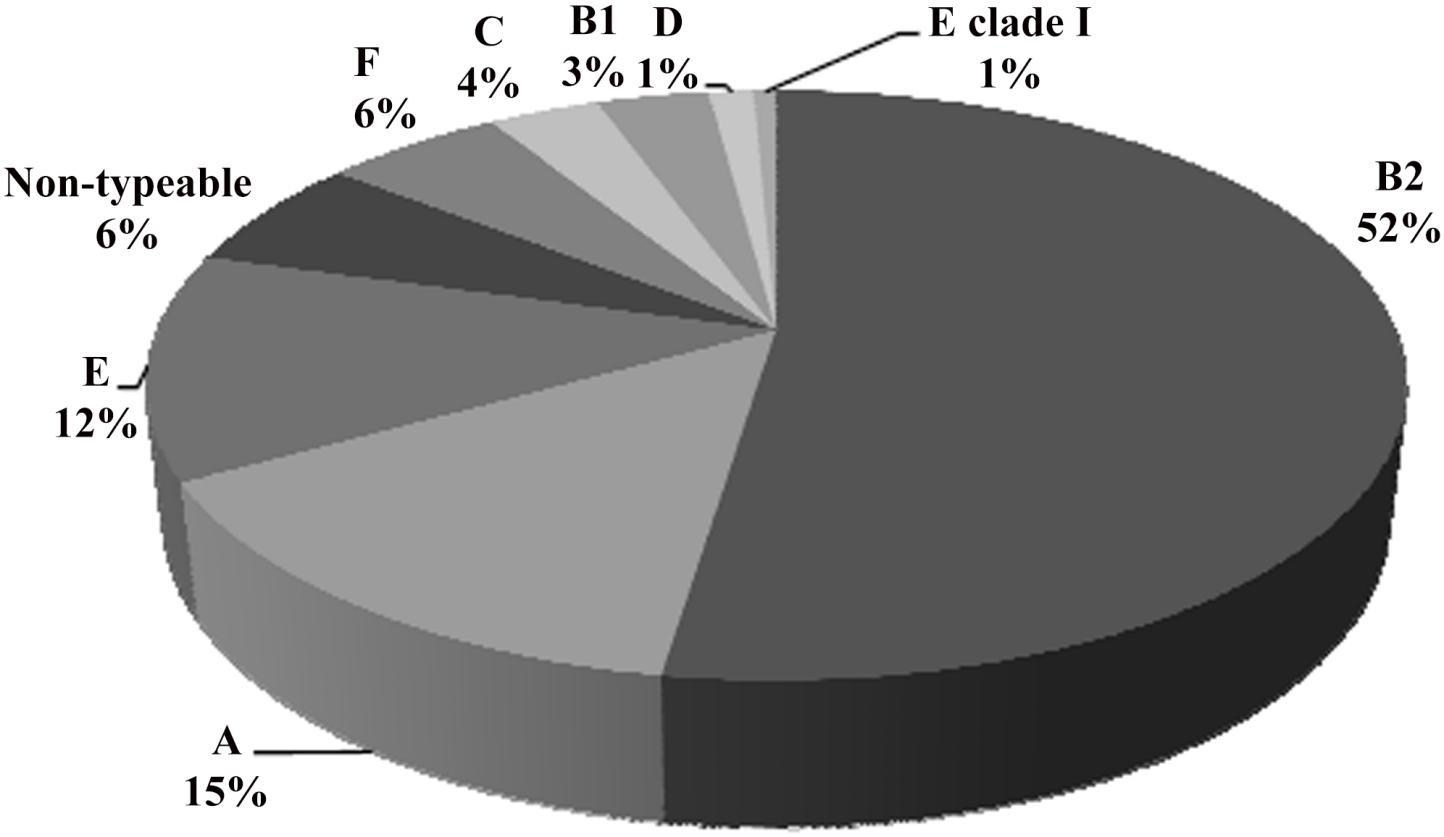
Distribution of phylogenetic groups among the isolates.

Seventy-six (52%) of all the isolates belonged to the B2 group and to a lesser extent to groups A and E (15% and 12%, respectively) (Fig 3). The presence of the other phylo-groups was lower, being 21% of the remaining phylo-groups. Significant differences were found when phylo-group B2 was compared with all the others separately (p<0.01 in all cases), but no differences were observed between VEC and obstetric IEC isolates.

### Relationship between the phylogenetic group, virulence factors and antimicrobial resistance

Statistically significant differences were found in the presence of VFGs among isolates belonging to phylogenetic group B2, being *hly*A (p<0.0001), *cnf*1 (p<0.0001), *pap*A (p = 0.003), *pap*C (p = 0.011), *foc*G (p = 0.002), *hra* (p = 0.009), *fyu*A (p<0.0001) and *ibe*A (p = 0.001) the most representative of this group regarding all the isolates, whereas *iut*A and *iuc*C were more frequently observed among the non-B2 groups (Table 3).

**Table 3:** Prevalence of virulence factor genes (VFGs) among the phylogenetic groups and the type of isolates according to their ampicillin resistance.

			Ampicillin Resistance^e^			
	Phylogenetic group		VEC^b^ isolates		Obstetric IEC^c^ isolates	
VFGs^a^	B2	Others^d^	AMP-S	AMP-R	AMP-S	AMP-R
	(n = 75)	(n = 70)	(n = 31)	(n = 51)	(n = 44)	(n = 19)
*hly*A	29 (39)**	**2 (3)**	**1 (3)**	18 (35)**	8 (18)	4 (21)
*cnf*1	31 (41)**	**3 (4)**	**1 (3)**	17 (33)**	11 (25)	5 (26)
*sat*1	27 (36)	29 (41)	11 (35.5)	18 (35)	16 (36)	11 (58)
*papa*	35 (47)**	**16 (23)**	7 (23)	16 (31)	16 (36)	12 (63)
*pap*EF	24 (32)	14 (20)	**3 (10)**	18 (35)**	12 (27)	6 (32)
*pap*C	30 (40)**	**14 (20)**	5 (16)	15 (29)	15 (34)	9 (47)
*foc*G	12 (16)**	**1 (1)**	1 (3)	8 (16)	2 (4.5)	2 (10.5)
*Hra*	34 (45)**	**17 (24)**	**5 (16)**	24 (47)**	15 (34)	7 (37)
*fyu*A	71 (95)**	**43 (61**)	**19 (61)**	47 (92)**	33 (75)	15 (79)
*iut*A	34 (45)	43 (61)	12 (39)	25 (49)	28 (34)	12 (63)
*iro*N	33 (44)*	**19 (27)**	**3 (10)**	26 (51)**	17 (39)	6 (32)
*iuc*C	**34 (45)**	46 (66)*	13 (42)	28 (55)	30 (68)	9 (47)
*ibe*A	14 (19)**	**1 (1)**	5 (16)	8 (16)	2 (4.5)	0

^a^VFGs: Virulence Factor Genes

^b^VEC isolates: Vaginal *Escherichia coli* isolates

^c^Obstetric IEC isolates: Obstetric Infection *Escherichia coli* isolates

^d^Others: A, E, Non-typeable, F, C, B1, D and E clade 1

^e^Ampicillin resistance: AMP, ampicillin; S, Susceptible; R, Resistant.

*p-value<0.05,

**p-value≤0.01

Ampicillin-resistant VEC isolates showed a greater number of VFs in comparison with ampicillin-susceptible isolates, being highly significant for the *hly*A (p = 0.001), *cnf*1 (p = 0.001), *pap*EF (p = 0.01), *hr*a (p = 0.005), *fyu*A (p = 0.001), and *iro*N (p<0.001) genes. However, VFGs tended to be more frequent among ampicillin-susceptible obstetric IEC isolates. Iron acquisition systems, such as *fyu*A and *iro*N, were also significantly more prevalent among tetracycline-resistant VEC isolates than their susceptible counterparts (*fyu*A: p = 0.023 and *iro*N: p = 0.008). However, tetracycline-resistant isolates from obstetric infection showed fewer VFs, being significant for *hly*A (p = 0.009), *cnf*1 (p = 0.019), *pap*A (p = 0.005) and *pap*C (p = 0.014). In addition, the aerobactin synthesis gene *iuc*C was significantly more frequently found among the tetracycline, trimethoprim/sulfamethoxazole, gentamicin and chloramphenicol-resistant isolates in comparison with the susceptible isolates. No highly significant differences were found in the distribution of the phylotype and antimicrobial agents.

## Discussion

The prevalence of vaginal *E*. *coli* found in this study was 13% similar to results of previous studies [17,18], but lower than in others [19]. In addition, the noteworthy percentage of maternal *E*. *coli* carriers suggests that vaginal *E*. *coli* colonization is a risk factor for pPROM and preterm birth [20,21] as well as the starting point for progression to severe infection [11].

A review of the current empiric treatment may be necessary, considering the high prevalence of ampicillin and gentamicin resistance in *E*. *coli* isolates collected from women with postpartum endometritis and intraamniotic infection, in agreement with previous reports [18], [22]. Therefore, the use of cephalosporines as first-line therapy for the treatment of mothers and neonates may be effective due to the high percentages of susceptibility [7], [23] and good outcomes shown in the treatment of peripartum sepsis [24]. Nevertheless, the geographical area should also be taken into account because of the particular increase in infections due to ESBL-producing organisms worldwide [18], [25], especially in developing countries [26].

The high presence of iron acquisition systems among the isolates was notable, with yersiniabactin (*fyu*A) being the most prevalent VFG among all the isolates (VEC: 80%, obstetric IEC: 76%), in contrast with earlier obstetric IEC isolates from the same hospital (only 18%) [22]. The direct links to iron-limited environments [27] and virulence as well as invasiveness have previously been confirmed by studies in bacteraemic *E*. *coli* strains [6]. It has been demonstrated that iron obtained through iron-uptake systems, such as heme, aerobactin, yersiniabactin or siderophores is necessary in UTIs and these systems show specific functions depending on the anatomical site [28] and may be targets for interventions, such as vaccines [29,30].

Higher percentages of genes associated with pathogenicity islands (PAIs), such as the *hly*A, *cnf*1 and *pap-* genes [31], have been observed among isolates from skin and soft tissue infections (SSTIs) [7], causing neonatal sepsis [32] as well as from patients with different sources of bacteraemia [8]. The relatively low frequency of these genes in our study is similar to that of VEC from East Japan, but lower than in strains from West Japan [1], United States [33], Slovenia [34] or Barcelona [17], suggesting the heterogeneity of *E*. *coli* strains to geographical area. However, the prevalence of the toxin *cnf*1 was higher than that found in previous obstetric IEC isolates from our hospital (25% *vs*. 9%) [22]. In contrast to other studies [1], [8], [32], [35], the least prevalent VFGs were *foc*G and *ibe*A, being found in less than 10% of obstetric infection isolates. Unlike previous obstetric IEC isolates, isolates causing sepsis presented the same percentage of the invasin *ibe*A than VEC in this study [22].

Similar to the results of other studies, more than half of the isolates in the present study belonged to virulence group B2 [11], [17]. The relevant statistically percentage of strains belonging to group A considerably differed from that reported by Iranpour et al. [12]. Surprisingly, group E and non-typeable strains were frequently observed compared to the results of Clermont et al. [13], which may be due to the great diversity, the extreme peculiarity or a recombination between phylo-groups among *E*. *coli* strains.

Group B2 presented the highest number of VFs among all the strains, in agreement with other studies performed in strains isolated from extraintestinal sites [5], [7], [36]. In contrast to prior findings, ciprofloxacin-resistant strains did not belong to non-B2 phylogenetic groups and neither did they show a loss of VFs related to PAIs [7], [37–39] However, the lower presence of these VFGs among tetracycline-resistant isolates causing obstetric infections was concordant with the results of Soto et al. [39]. Furthermore, the selection of ampicillin-resistant strains presenting high rates of VFs has been previously shown by Petkovsek et al. [7]. Regardless of the ecological and host-dependent factors with respect to the severity of infection [6], [34], these strains have been shown to cause obstetric and neonatal infections and may have a serious clinical impact [4], [40].

The present study has some limitations since data on characterization of vaginal and obstetric infections *E*. *coli* strains are scarce, and many comparisons are made using other types of extraintestinal isolates. However, besides the virulence profile of VEC resembling that of ExPEC isolates, our findings are useful to generate relevant hypotheses on this little-known topic because of the relationship of a wide range of VFs and antimicrobial agents with the new phylogenetic analysis.

In conclusion, vaginal *E*. *coli* colonization may be a risk factor for complications during pregnancy, especially if these strains are ampicillin-resistant and show many virulence factors resulting in treatment failure or infections. Therefore, it is important to know the resistance and virulence associated with *E*. *coli* strains from different anatomic sites [2] in order to determine the probability and the severity of infection these strains may cause. The characterization of *E*. *coli* isolates which colonize the vagina and cause obstetric infections may be especially of help to develop interventions, and avoid the causal link between maternal carriage and obstetric and subsequent puerperal infections.
